# Substance Use Among Residents of Homeless Shelters During the COVID-19 Pandemic: Findings From France

**DOI:** 10.3389/ijph.2022.1604684

**Published:** 2022-08-25

**Authors:** Honor Scarlett, Maria Melchior, Camille Davisse-Paturet, Tarik El. Aarbaoui, Cécile Longchamps, Natasha Figueiredo, Simon Ducarroz

**Affiliations:** ^1^ Sorbonne Université, INSERM, Institut Pierre Louis d’Epidémiologie et de Santé Publique, Paris, France; ^2^ CNRS, Institut Convergences Migration, Aubervilliers, France; ^3^ Research on Healthcare Performance RESHAPE, INSERM U1290, Université Claude Bernard Lyon 1, Lyon, France

**Keywords:** mental health, COVID–19, substance use, homelessness, migrant, France

## Abstract

**Objectives:** To record the prevalence and risk factors of substance use amongst homeless persons during the COVID-19 pandemic.

**Methods:** The ECHO study consisted in two independent cross-sectional waves of data collection in the regions of Paris, Lyon, and Strasbourg during the Spring of 2020 (*n* = 530) and 2021 (*n* = 319). Factors associated with substance use were explored using generalised logistic regression models.

**Results:** The most prevalent substance used was tobacco (38%–43%), followed by alcohol (26%–34%). The use of both substances positively associated with each other, although risk factors varied depending on the substance. The only factors consistently associated with alcohol and tobacco use were being male, exposure to theft/assault and participants’ region of origin. Whilst the rate of tobacco use was relatively stable between Spring 2020 and 2021, alcohol use was more common in 2021.

**Conclusion:** These findings highlight a high prevalence of substance use amongst homeless persons. People experiencing homelessness face specific challenges in the context of the pandemic, alongside greater vulnerability to illness and low healthcare access, therefore the need to improve prevention and support services for substance abuse within this population is vital.

## Introduction

Evidence suggests that the COVID-19 pandemic has significantly impacted rates of substance use in the general population [1–4]. However, these changes are complex and ongoing. On the one hand, environmental stress is a known risk factor for substance use disorders [5], with increases in alcohol and tobacco consumption recorded during natural disasters [6, 7] terrorist incidents [8, 9] and humanitarian conflicts [10, 11]. The stress of the COVID-19 pandemic, alongside associated increases in social isolation [12], may have therefore contributed to increasing rates of substance use [13]. COVID-19 preventative measures, such as lockdowns, staff shortages and reduced opening hours, were also predicted to disrupt access to support and drug services [14–16]. Nevertheless, the pandemic is likely to have decreased drug availability, due to reduced drug trafficking and market shortages [17], alongside reducing opportunities for consumption, with users themselves having less freedom of movement, disrupted income and fewer social interactions. For these reasons, the effect of the pandemic on substance use warrants investigation.

One of the populations most vulnerable to substance use is those without stable housing. Government estimates found the number of homeless people in Metropolitan France rose from 93,000 in 2001 to 141,500 in 2012 [18]. And within the Paris area, an estimated 29% of homeless persons suffer from addiction, with 18% of those surveyed regularly consuming at least one illegal substance [19]. In parallel, rates of frequent smoking are significantly higher amongst homeless persons (∼84%) than in the French general population (∼25%) [20, 21]. Considering that smoking is the leading cause of preventable death in Europe [22–24], associated strongly to social inequalities, examining the extent to which patterns of tobacco use have evolved in the context of the COVID-19 pandemic among homeless groups is of public health interest. Not only are homeless persons more susceptible to tobacco-related illness and subsequent fatality [25–27], but the financial burden of addiction may also prolong financial insecurity and therefore housing instability [22].

Many of the factors explaining the association between homelessness and substance use are likely to have been impacted by the COVID-19 pandemic. Not only are periods of homelessness often accompanied by increased stress, but fewer supporting factors remain. Financial difficulties, increased isolation and social stigma all inhibit resilience, leaving those experiencing homeless increasingly vulnerable to addiction. Studies have shown that housing instability and food insecurity increased the risk of turning to substance use to cope with depression and anxiety during the pandemic [1, 28]. Moreover, government policies surrounding housing have greatly fluctuated since the initial outbreak of COVID-19, and homeless persons have had to recurrently adapt. Qualitative studies have shown that, whilst temporary shelters created as a COVID-19 preventative measure may have had an initially positive effect on addictive substance use, the subsequent eviction of homeless persons resulted in them either resuming or increasing their drug consumption [29]. Findings taken from multiple time-points throughout the duration of the pandemic are therefore necessary to both understand the permanence of its impact and help inform public health measures.

Here we present data on the prevalence and associated risk factors for tobacco and alcohol use amongst persons experiencing homelessness during the COVID-19 pandemic. Importantly, the ECHO study spans two distinct periods: the onset of the pandemic (Spring, 2020) and the following Spring (2021). Through this, we were able to collect data from persons sleeping rough as well as those living in shelters, thereby providing valuable information on a group for which evidence regarding the experience of the COVID-19 pandemic is currently limited.

## Methods

### Study Design

The ECHO study is a cross-sectional investigation conducted amongst persons without stable housing in France. The findings presented here are based on two waves of data collection, taken during the Spring of 2020 (02/05/20–07/06/20) (W1) and 2021 (09/03/21–31/05/21) (W2). Whilst the samples for each wave were distinct, the methodology was generally consistent between the two. Participants were recruited from shelters located in the regions of Paris (W1 *n* = 12; W2 = 9), Lyon (W1 = 5; W2 = 10) and Strasbourg (W1 = 1). Recruitment for W2 also incorporated day centres (*n* = 7), allowing the inclusion of those sleeping rough. Interviews were conducted both in person (W1 = 98%; W2 = 100%) or by telephone (W1 = 2%), in French, English or participants’ chosen language, with the help of independent interpreters, to minimise bias due to language barriers (W1 = 33%, W2 = 13%). Over 20 languages were used, most frequently Arabic, Pashto and Dari. Participants were excluded if aged under 18 years, significantly inebriated or presenting cognitive disorders that prevented consent. The study protocol was approved by the Ethical Research Committee of the University of Paris (CER-2020-41).

### Assessment of Substance Use

The assessment of both substance use and risk factors was done via direct, one-on-one interviews with participants. All questions were based on a pre-determined, quantitative questionnaire, designed in each study wave by the ECHO research team. Whilst the questionnaire could have been self-administered, interviews were conducted to reduce exclusion based on participants’ language or literacy. In both study waves, participants were asked to report their current use of tobacco and alcohol on a 3-point scale ranging from “every day,” “sometimes”; or “no”. For multivariate analyses, “every day” and “sometimes” were combined, generating two categories for current use (yes/no). As a secondary objective of this study, participants also reported cannabis use on the same scale, however, due to its illegality, a “does not wish to answer” option was available in W1. For W2, questions on substance use were exclusive to the supplementary questionnaire, and refusal to answer was registered as missing data.

### Risk Factors

Factors considered potentially relevant to patterns of substance use were the following: age (18–29; 30–49; 50 + years), sex (male; female), partnership status (stable partner; single), family status (no children; living with children; has children but living separately), educational level (no school/incomplete primary education; primary/high school education; College/higher education), employment (none; only before lockdown; both before and during lockdown), duration of stay in France (<1 year; 1–5 years; 5+ years, including French natives), French language aptitude (low; moderate/fluent), administrative status (French native; residence permit holder; asylum seeker; no residence permit; other), health insurance (yes; no), chronic illness (yes; no), food insecurity (yes; no), feelings of safety (yes; no), exposure to theft or assault during the first lockdown (yes; no), contact with friends/family (yes; no), and participants’ previous accommodation (centre/association; unestablished shelter e.g., camps, squats; street; friends/family/other).

French language aptitude was calculated from the sum score of self-reported French speaking, reading and writing ability, each rated on a 4-point Likert scale. Participants’ degree of loneliness was measured based on the UCLA loneliness scale [30]. Health literacy was calculated as a sum of participants’ scores to either the fifth (W1) or fourth and seventh (W2) dimensions of the Health Literacy Questionnaire [31], with the threshold between low and high set as an average agreement of ≤50% for W1 and ≤33% for W2. Depression was assessed via the nine-item Patient Health Questionnaire (PHQ-9) in W1 (cut-off score 10 [32]), and the four-item PHQ-4 in W2 (cut-off score 5). Both are validated for use in multicultural settings [33, 34]. Finally, participants region of origin was divided into France; Europe excluding France; the Middle East; North Africa; Sub-Saharan Africa; and Other (categories defined in Supplementary Table S1).

### Data Analyses

To identify factors associated with alcohol and tobacco use, the following approach was implemented. First, differences in the prevalence of substance use between Spring 2020 and 2021 were examined using Chi-square (X^2^) analysis. We then tested associations with factors considered relevant *a priori*, in relation to substance use, using generalised logistic regression models, implementing separate models for each study wave. Missing covariate values were imputed using Multivariate Imputation by Chained Equations (MICE) [35].

Participants from American, South-East Asian, and Western Pacific regions were excluded due to insufficient sample size (W1 *n* = 5; W2 = 12). Variables included in multivariate statistical models were determined *via* univariate X^2^ analyses, as supported by Hosmer and Lemeshow [36, 37]. An 80% confidence limit was used [38], to prevent the arbitrary exclusion of important variables [39, 40]. For measures of prevalence, cannabis use was also included, allowing us to further investigate interactions between respective substances.

All data analyses were performed on R Version 4.1.1 [41, 42].

## Results

### Sample

Overall, 1564 persons were invited to take part in the ECHO study. Following participant refusal or unavailability, 535 participants were interviewed in Spring 2020, of which 530 had sufficient data on substance use, and 523 in Spring 2021, of which 319 had sufficient data on substance use. Demographic information on both study samples is available in Table 1.

**TABLE 1:** Demographic characteristics of study participants. All *p* values shown are based on Chi-Square (X^2^) analysis. (France, 2020–2021).

	Spring 2020 (*n* = 530)	Spring 2021 (*n* = 319)	*p*
Sex			
Male	75% (377)	67% (215)	*
Female	25% (126)	33% (104)	
Age range (years)			
18–29	43% (218)	23% (73)	***
30–49	42% (209)	49% (155)	
50+	15% (76)	28% (90)	
Partnership status			
Yes, has a stable partner	37% (178)	29% (87)	*
No stable partner	63% (306)	71% (209)	
Family status			
No children	49% (230)	46% (146)	0.13
Has children, but not living with them	30% (143)	37% (117)	
Currently living with their children	21% (99)	17% (55)	
Highest education level			
No school or incomplete primary education	29% (143)	14% (44)	***
Primary or high school education	54% (269)	57% (181)	
College or higher education	17% (82)	29% (93)	
Employment status			
Unemployed	73% (360)	79% (254)	N/A
Employed before lockdown	20% (97)	21% (66)	
Employed before and during lockdown	7% (35)		
Perceived food insecurity			
Not food insecure	62% (307)	43% (139)	***
Food insecure	38% (191)	57% (181)	
Accommodation before current centre			
Other centre/charity	20% (99)	18% (59)	***
Unestablished shelter/squat	24% (119)	2% (6)	
Street	41% (207)	73% (234)	
Friends/family/other	16% (78)	7% (21)	
Time since arrival to centre			
Less than 1 month	53% (260)	—	N/A
1 month–1 year	29% (142)	—	
Over 1 year	18% (89)	—	
Region of birth			
French native	11% (57)	16% (52)	***
Europe (other than France)	11% (59)	8% (25)	
Middle East^a^	35% (185)	10% (33)	
Northern Africa^b^	10% (55)	19% (62)	
Sub-Saharan Africa	31% (162)	45% (143)	
Other	2% (12)	2% (5)	
Administrative status			
French native	11% (56)	16% (52)	***
Residence permit	23% (117)	30% (97)	
Asylum seeker	31% (156)	5% (16)	
No residence permit	26% (128)	43% (139)	
Other	8% (42)	5% (16)	
Health status			
Chronic illness (no)	74% (370)	47% (150)	***
Chronic illness (yes)	26% (127)	53% (167)	
Healthcare			
Medically insured/covered^c^	68% (343)	87% (278)	***
Uninsured	32% (159)	13% (40)	
Health literacy			
Low	15% (71)	—	N/A
High	85% (396)	—	
Symptoms of depression			
No	70% (352)	69% (220)	0.67
Yes	30% (148)	31% (100)	
French aptitude (self-reported)			
Low	54% (286)	10% (33)	***
Moderate /Fluent	46% (239)	90% (286)	
Trusts government information on COVID-19			
Yes	78% (365)	63% (185)	***
No	22% (100)	38% (111)	
Duration of stay in France			
<1 year	43% (222)	20% (63)	***
1–5 years	31% (159)	29% (92)	
5 + years	27% (139)	51% (163)	
Loneliness			
Not lonely, both now and before	32% (161)	22% (93)	***
As lonely as before	15% (76)	17% (69)	
More lonely than before	37% (186)	30% (126)	
Less lonely than before	15% (77)	31% (130)	
Social contact			
In regular contact with friends and family	88% (435)	—	N/A
No contact with friends and family	12% (62)	—	
Safety			
Has felt safe since lockdown	71% (354)	—	N/A
Has felt unsafe since lockdown	29% (147)	—	
Exposure to assault			
No theft or assault since lockdown	88% (438)	—	N/A
Theft or assault since lockdown	12% (58)	—	

a Middle Eastern countries relevant to this sample: Afghanistan, Iran, Iraq, Pakistan, Palestine, Saudi Arabia, Somalia, Sudan.

b North African countries relevant to this sample: Algeria, Libya, Morocco, Tunisia.

c Including State Medical Assistance (AME) for undocumented migrants.

### Rates of Substance Use

Rates of substance use are shown in Table 2. Tobacco was the most commonly used substance in both study waves (42% of participants in Spring 2020; 38% in 2021), followed by alcohol (26%; 34%), and cannabis (10%; 9%). Univariate analyses found tobacco use to be significantly associated with the use of both alcohol (*p < 0.001*) and cannabis (*p < 0.001*) in both waves. Alcohol use also significantly associated with cannabis use (*p < 0.001*)*.*

**TABLE 2:** Rates of tobacco, alcohol, and cannabis use amongst study participants during the COVID-19 pandemic during Spring 2020 (*n* = 530) and 2021 (*n* = 319). All *p* values shown are based on univariate regression analysis. (France. 2020–2021).

	Tobacco % (n)		Alcohol % (n)		Cannabis % (n)	
	Spring 2020	Spring 2021	Spring 2020	Spring 2021	Spring 2020	Spring 2021
Total users Of which was used…	42% (223)	38% (120)	26% (136)	34% (109)	10% (55)	9% (30)
In isolation	48% (106)	40% (48)	31% (42)	41% (45)	4% (2)	0
With tobacco	REF	REF	69% (94) (*p < 0.001*)	57% (62) (*p < 0.001*)	95% (52) (*p < 0.001*)	93% (28) (*p < 0.001*)
With alcohol	42% (94) (*p < 0.001*)	52% (62) (*p < 0.001*)	REF	REF	55% (30) (*p < 0.001*)	67% (20) (*p < 0.001*)
With cannabis	23% (52) (*p < 0.001*)	23% (28) (*p < 0.001*)	22% (30) (*p < 0.001*)	18% (20) (*p < 0.001*)	REF	REF

**FIGURE 1:**
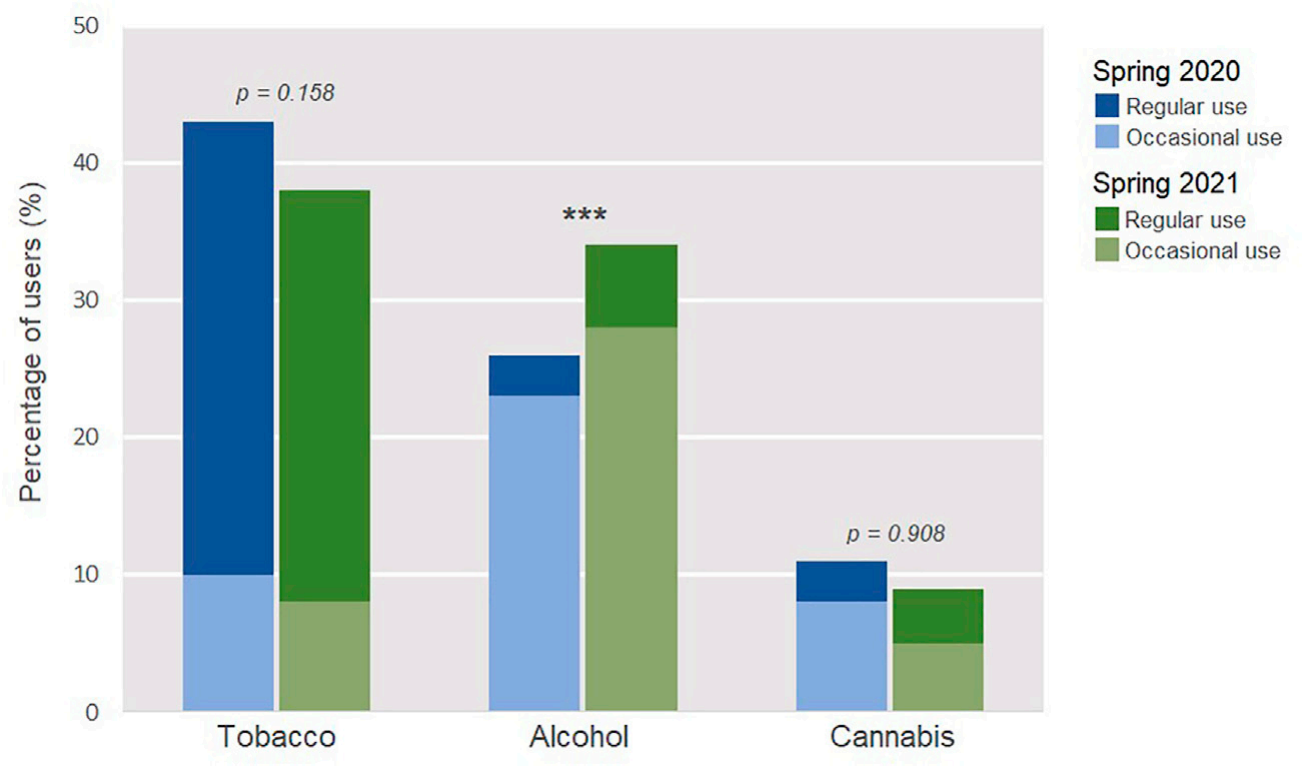
Rates of tobacco, alcohol and cannabis use amongst homeless persons interviewed in the Spring of 2020 (*n* = 530) and 2021 (*n* = 319). Chi-Square (X^2^) analysis. (France. 2020–2021).

Differences between rates of substance use between the two study waves are shown in Figure 1. When compared to 2020, alcohol use was more prevalent in 2021 (*p < 0.001*)*.*

### Risk Factors of Substance Use

Factors associated with alcohol and tobacco use, determined via multivariate regression, are shown in Table 3. In Spring 2020, characteristics associated with tobacco use were being male (aOR:5.04; 95% CI: 2.43–10.46), not having a stable partner (aOR:2.01; 95% CI: 1.16–3.46), being medically uninsured (aOR:2.10; 95% CI: 1.21–3.64) and exposure to theft or assault (aOR:2.55; 95% CI: 1.32–4.93). Compared to French natives, participants born in the Middle East (aOR:0.43; 95% CI: 0.21–0.88) or Sub-Saharan Africa (aOR:0.22; 95% CI: 0.11–0.41) were less likely to smoke tobacco, whilst those born in other European countries were more likely (aOR:4.54; 95% CI: 2.03–10.14). Participants who had lived in France for more than 5 years were also more likely to smoke than those living in France for less than 1 year (aOR:2.34; 95% CI: 1.03–5.31). In Spring 2021, tobacco use associated with being male (aOR:4.42; 95% CI: 2.09–9.37) and medically uninsured (aOR:2.88; 95% CI: 1.09–7.64).

**TABLE 3:** Risk factors of tobacco and alcohol use amongst study participants. Multivariate, logistic regression models (aOR, 95% CI). (France. 2020–2021).

		Tobacco				Alcohol			
		Spring 2020^a^		Spring 2021^b^		Spring 2020^a^		Spring 2021^b^	
		aOR	95% CI	aOR	95% CI	aOR	95% CI	aOR	95% CI
Sex	Female	1		1		1		1	
	Male	**5.04**	**2.43–10.46**	**4.42**	**2.09–9.37**	**3.44**	**1.63–7.25**	**2.53**	**1.26–5.07**
Partnership status	Yes, has a stable partner	1		1		1		1	
	No stable partner	**2.01**	**1.16–3.46**	1.67	0.77–3.61	1.06	0.61–1.85	0.96	0.46–2.01
Family status	No children	1		1		1		1	
	Has children, but not living with them	0.97	0.53–1.80	1.14	0.52–2.50	1.31	0.72–2.38	1.07	0.50–2.29
	Currently living with their children	0.59	0.24–1.45	0.63	0.21–1.84	1.00	0.41–2.45	0.65	0.24–1.78
Highest education level	No school/incomplete primary education	1		1		1		1	
	Primary or high school education	1.29	0.76–2.19	2.24	0.85–5.95	1.15	0.66–2.02	**3.80**	**1.44–10.00**
	College/Higher education	0.82	0.39–1.74	1.51	0.54–4.22	1.47	0.69–3.14	2.33	0.84–6.47
Age range (years)	18–29	1		1		1		1	
	30–49	1.33	0.78–2.28	1.10	0.49–2.47	1.09	0.64–1.86	1.87	0.86–4.08
	50+	0.67	0.28–1.62	1.72	0.66–4.50	0.55	0.22–1.36	1.59	0.63–4.01
Experiencing food insecurity	No	1		1		1		1	
	Yes	1.01	0.63–1.62	1.23	0.66–2.29	1.28	0.79–2.07	0.81	0.45–1.46
Loneliness	Not lonely (before or after)	1		1		1		1	
	As lonely as before	1.17	0.58–2.38	0.97	0.40–2.37	1.23	0.61–2.48	1.14	0.49–2.68
	More	1.00	0.56–1.79	0.98	0.45–2.12	0.99	0.54–1.81	1.46	0.71–3.03
	Less	0.99	0.48–2.04	0.84	0.26–2.67	1.02	0.49–2.16	1.27	**0.42–3.82**
Region of birth	French native	1		1		1		1	
	Europe (other than France)	**4.54**	**2.03–10.14**	0.95	0.35–2.59	1.41	0.68–2.93	0.66	0.24–1.76
	Middle East^c^	**0.43**	**0.21–0.88**	0.79	0.33–1.90	0.91	0.46–1.82	0.61	0.24–1.56
	North Africa^d^	0.88	0.43–1.83	1.40	0.65–3.00	0.63	0.29–1.37	**0.24**	**0.11–0.55**
	Sub-Saharan Africa	**0.22**	**0.11–0.41**	**0.26**	**0.13–0.52**	1.11	0.60–2.03	0.75	0.39–1.45
Time since arrival to France	Less than 1 year	1		1		1		1	
	1–5 years	1.71	0.95–3.09	0.70	0.26–1.91	1.07	0.59–1.92	1.25	0.46–3.42
	5+ years	**2.34**	**1.03–5.31**	1.19	0.44–3.23	1.11	0.48–2.56	1.83	0.67–5.00
Healthcare	Medically insured/covered^e^	1		1		1		1	
	Uninsured	**2.10**	**1.23–3.60**	**2.88**	**1.09–7.64**	1.11	0.65–1.92	1.34	0.53–3.34
Symptoms of depression	no	1		1		1		1	
	Yes	1.07	0.64–1.80	1.26	0.66–2.42	**1.66**	**1.00–2.75**	0.77	0.41–1.45
Administration status	French native	1		1		1		1	
	Residence permit	0.59	0.32–1.08	0.88	0.44–1.78	1.01	0.55–1.83	0.69	0.35–1.35
	Asylum seeker	0.87	0.45–1.66	0.81	0.21–3.04	0.92	0.49–1.75	0.28	0.06–1.26
	No residence permit	0.89	0.46–1.72	0.59	0.32–1.09	0.81	0.42–1.56	0.62	0.34–1.15
	Other	0.82	0.37–1.83	0.66	0.20–2.15	1.19	0.55–2.60	0.62	**0.19–2.03**
Trusts government information on COVID-19	Yes	1		1		1		1	
	No	1.38	0.82–2.33	1.02	0.56–1.85	1.40	0.83–2.36	1.44	0.81–2.57
Exposure to assault	No theft or assault since lockdown	1		1		1		1	
	Theft or assault since lockdown	**2.55**	**1.32–4.93**	—	—	**1.83**	**1.01–3.31**	—	
Accommodation before current	Other organised center	1		1		1		1	
	Unestablished shelter	0.55	0.28–1.10	0.43	0.07–2.53	0.81	0.41–1.63	0.23	0.04–1.43
	Street	0.59	0.31–1.11	0.86	0.41–1.81	0.77	0.40–1.46	0.83	0.39–1.75
	Friends/family/other	0.60	0.27–1.31	0.35	0.09–1.40	1.01	0.47–2.16	0.98	0.25–3.84

Values with statistical significance shown in bold.

a *n* = 530.

b *n* = 319.

c Middle Eastern countries relevant to this sample: Afghanistan, Iran, Iraq, Pakistan, Palestine, Saudi Arabia, Somalia, Sudan.

d North African countries relevant to this sample: Algeria, Libya, Morocco, Tunisia.

e Including State Medical Assistance (AME) for undocumented migrants.

Characteristics associated with alcohol use in Spring 2020 were being male (aOR:3.44; 95% CI: 1.63–7.25), exposure to theft or assault (aOR:1.83; 95% CI: 1.01–3.31) and experiencing symptoms of depression (aOR: .66; 95% CI: 1.00–2.75). In Spring 2021, alcohol use associated with being male (aOR:2.53; 95% CI: 1.26–5.07) and having had primary or high school education (aOR:3.80; 95% CI: 1.44–10.00) as opposed to no schooling. Compared to French natives, North African participants were less likely to drink alcohol (aOR:0.24; 95% CI: 0.11–0.55).

## Discussion

This study aimed to describe the use of psychoactive substances amongst persons with unstable housing during the course of the COVID-19 pandemic. Participants were most likely to use tobacco (38–43%), followed by alcohol (26–34%). The only factor consistently associated with use of both tobacco and alcohol in both waves was being male. Tobacco use was also associated with exposure to theft or assault during the first lockdown, being medically uninsured, living in France for over 5 years, and not having a stable partner. For alcohol use, risk factors were exposure to theft or assault, having symptoms of depression and having primary or secondary school education (as opposed to no schooling or incomplete primary education). Participants migrating from Europe were also more likely to smoke tobacco, whilst those born in the Middle East or Sub-Saharan Africa significantly less, and those from North Africa were less likely to drink alcohol.

### Rates of Substance Use

Tobacco was the most frequent substance used in both ECHO study waves. Cross-sectional data on the French general population during Spring 2020 saw 21% were tobacco smokers (occasional and regular) [4]. Our findings then suggest a rate amongst homeless persons roughly double that of the general population. Considering that homeless persons present higher rates of medical comorbidities [25, 26, 45] and are therefore more at risk of tobacco-related illness or fatality [26, 46], these findings are cause for concern. Additionally, despite universal health care, socioeconomically disadvantaged groups are less likely to have access to smoking cessation programs, leading to large inequalities in smoking which appear to have increased in the context of the COVID-19 pandemic [47]. Regarding alcohol, rates within ECHO were also higher than national levels (17% regular drinkers) [48].

### Differences in Behaviour Across the Pandemic

Whilst the rates of tobacco use were similar between Spring 2020 and 2021 (38%–43%), alcohol use was more frequent amongst participants in 2021 (34%) than 2020 (26%). Several key factors may account for the higher rate of alcohol use seen in Spring 2021; first, during the initial lockdown period, restrictions may have decreased the availability of alcohol, both physically (bar/shop closures; exit permits) and financially (reduced hours; job loss) [17]. Many of these restrictions were either lifted or relaxed by Spring 2021, during which France experienced its third lockdown. This can be seen, for example, in the rate of employment. Among the ECHO population surveyed in Spring 2020, 93% were unemployed, compared to just 79% in Spring 2021. Whilst employment status did not associate with alcohol use in our study population, nor amongst non-homeless populations during the pandemic [49], factors associated with fewer restrictions (such as increased socialisation and disposable income) may have enabled more frequent drinking [50]. The higher rates of alcohol use amongst those surveyed in 2021 may also be specific to each groups’ respective living situation. All participants in the first wave were housed in temporary shelter, which may have had a positive influence on alcohol consumption. A stable, communal living environment can provide greater support than is often available for those sleeping rough, alongside that from social workers specifically hired by the organisations themselves [51]. On-site case-management and social support, given through the Housing First initiative, has been shown to reduce alcohol-use amongst previously-unsheltered homeless persons [52, 53]. Furthermore, qualitative findings collected during April–August 2020 show homeless-centre users found support services acted as a ‘lifeline’ during the pandemic, providing invaluable stability during a time of such disruption [54]. In comparison, the ECHO sample interviewed in Spring 2021 had a variety of living situations. Alongside a reduced level of support, this may elevate rates of alcohol use bidirectionally: those who drink may struggle more to find or maintain shelter, whilst those without temporary shelter may then be more likely to drink [55] Moreover, the discontinuation of the housing services initially provided in response to the pandemic (of which the first-wave participants benefitted) may have triggered alcohol consumption. In support of this, Scallan et al. [29] found disruptions to emergency housing, provided temporarily during the pandemic, resulted in increased addictive substance use. Together, our findings may then indicate the importance of a consistent, supportive living environment for the promotion of healthy behaviours, particularly following the fluctuations in housing caused by the pandemic.

Several of these factors, however, would also apply to tobacco, despite no significant difference being noted. Our findings also contrast with those from the general French population, which found tobacco smokers were more likely to report an increase in use (27%) since the onset of the pandemic, whilst alcohol use generally decreased—at least in frequency [52]. This may then indicate that alcohol drinking was a more popular coping mechanism for the progressive, long-term strain of the pandemic amongst homeless populations [54]. In support of this, drinking to cope during the pandemic was seen to associate with depression [49], for which the ECHO population showed higher-than-national levels [55]. Alcohol use within both study populations may have also depended more on social liberty (the ability to socialise; beg for money) and financial security than tobacco. In fact, several studies showed social isolation was not a significant risk factor for changing tobacco consumption during the pandemic, with one even finding it to be the most popular reason for decreasing cigarette use [56, 57].

### Factors Associated With Substance Use

#### Gender and Assault

Among risk factors of substance use examined in our study, being male was the only factor associated with both alcohol and tobacco use across both waves. Accordingly, cross-sectional data collected in 2009 from homeless persons in Paris found men were more likely to consume at least one recreational drug, and be dependent on alcohol [19]. In contrast, rates of smoking within the general French population show no significant difference between men and women [4], with Canadian findings also showing that the use of substances as a coping mechanism during the pandemic did not differ by gender [58]. This association may then be more specific to homeless persons. However, these results do not mean that homeless women are not vulnerable to substance abuse [59]. In our sample, the only other factor associated with both tobacco and alcohol use was an exposure to theft or assault. Whilst previous literature supports this [60], studies suggest the association between assault and substance use is stronger amongst women [61, 62]. Considering homeless women also present a far greater risk for assault than homeless men [63, 64], this link between substance use and assault should be kept as an important consideration for residents of women’s shelters. In particular, women are disproportionately affected by intimate partner violence (IPV) than men [65, 66]. This is particularly relevant, as not only are both poverty and homelessness risk factors for IPV, but rates have climbed significantly since the onset of the pandemic [65, 67].

#### Migrant Status

The lower rate of alcohol use observed amongst participants born in Northern Africa may result from the entirety of this group having migrated from Muslim-majority countries, where alcohol consumption is actively discouraged [68]. Nevertheless, reports have shown that migrants from Muslim-majority countries who do drink alcohol are more likely to engage dangerous drinking-behaviours [69], so these results should be interpreted with caution. For tobacco use, Middle Eastern or Sub-Saharan African participants showed significantly lower rates than French natives, whilst those born in Europe showed rates significantly higher. This is in line with pre-pandemic findings, which showed migrants from sub-Saharan Africa to be significantly less likely to smoke than French nationals [70]. This may also explain the association between smoking and the amount of time participants had spent in France; those who had lived in France for over 5 years were more likely to smoke than new arrivals (<1 year), therefore French natives may have partly driven these results.

Another explanation for the relationship between migrants’ region of origin and substance use may be the motives for migration. These are likely to depend on the region itself [71, 72], and may contribute differentially to one’s tendency to seek external coping mechanisms. For example, post-traumatic stress disorder (PTSD) is a known trigger for substance abuse [73], and is also established to result from experiences of migration [74]. Persons migrating due to war or domestic violence may then be at greater risk for addictive behaviours. In support of this, research has shown that levels of PTSD among homeless migrant mothers in Paris depended significantly on country of origin and motivation for departure [75].

Regardless, the higher rate of smoking seen amongst those migrating from Europe is still a cause for concern. Migrant populations have both lower rates of medical insurance and greater difficulties accessing medical care [76]. Moreover, a lack of medical insurance was also seen to be a risk factor for tobacco use in both waves. The COVID-19 pandemic is likely to have accentuated these inequalities further. Findings from the UK show the shift to more virtual healthcare during the pandemic exacerbated barriers to health-care access amongst migrants [77, 78]. Within homeless populations, those migrating from Europe may then be even more vulnerable to tobacco-related illness and mortality.

#### Mental Health

Symptoms of depression were associated with a higher likelihood of alcohol use during the early stages of the pandemic in our study. During this period, depression was also found to be associated with increased alcohol consumption in the general population [4]. Within the ECHO study sample, both waves showed rates of depression (30–31%) higher than national averages (∼20%) [79]. Serious mental disorders are known risk factors for homelessness, propagating the associated issues with employment and access to medical care and social support [80, 81]. Even pre-pandemic, a Parisian study found half of homeless persons surveyed with psychotic disorders were addicted to at least one drug, with 30% alcohol-dependent [19]. Moreover, 68% of ECHO study participants in Spring 2020 and 78% in 2021 reported feeling some degree of loneliness, an established long-term risk factor for depression [82, 83] and addictive behaviours [84]. These figures align with findings that loneliness increased during the COVID-19 pandemic especially amongst those with low socioeconomic status [85, 86].

### Wider Implications

These results may be useful to guide both the prevention and treatment of substance abuse in homeless populations following the COVID-19 pandemic. Existing preventative measures for addictive behaviour might not be effective; studies have shown that whilst homeless adults who have experienced more frequent homeless episodes have higher odds of receiving anti-smoking support, they show lower rates of quitting [26]. Moreover, several of the risk factors identified, such as exposure to assault and symptoms of depression, have also increased in prevalence during the pandemic [66, 67, 87, 88].

Whilst risk factors associated with substance use varied between tobacco and alcohol, the use of different substances strongly associated with each other. Research suggests many of the genetic and environmental vulnerabilities towards using different addictive substances are shared [89, 90], therefore the risk factors identified for tobacco and alcohol use within this sample may remain pertinent to other substances. Considering the concurrent use of multiple substances also increases the chance of subsequent injury [91], interventions considering all potential risk factors may be more successful in preventing substance-related morbidity and mortality.

### Limitations and Strengths

Our study has several limitations which need to be addressed. First, the primary focus of the ECHO study was not to investigate substance use. Therefore, our assessment was relatively limited, comprising of one multiple-choice measure per substance. Despite this, the more generalised questionnaire design allowed us to account for a wider range of variables in the limited interview time available, often not examined in relation to substance use. Secondly, ECHO comprises two cross-sectional waves of data collection, based on separate samples. This therefore limits our assessment of longitudinal patterns of substance use. The most significant difference between the two study waves was participants’ housing situation; whilst wave one included only persons living in temporary accommodation at the time of investigation, wave two recruited persons from a wider range of situations. However, it is important to note that during the Spring of 2020, France had an active policy of providing temporary shelter to all persons sleeping rough, in order to limit the propagation of COVID-19. This sample may thus be more reflective of the general homeless population than those typically residing in temporary accommodation. In support of this, 41% of wave one participants were living on the street before their current shelter, thereby indicating the inclusion of various experiences of homelessness.

Finally, this study may be biased due to our reliance on participants’ self-reports. In comparison to biological markers, self-reports are known to generate underestimations of substance use [43]. Moreover, within our study, both data collection points occurred during Ramadan, a month associated with abstinence from drugs within the Muslim community. Although no data on participants’ religious practice were collected, 61% of our sample originated from a predominantly Muslim country. If practising Islam, these participants are then potentially more likely to have both reduced their consumption during this period, and under-report their levels of use due to social or cultural desirability bias [44]. This tendency towards underestimation may have also been accentuated by the precariousness of our study population’s accommodation, as participants may have feared that reporting recreational drug use would have jeopardised their right to shelter. To counteract this, before every interview, participants were reminded that their responses were anonymous and would not impact their right to accommodation, and that they were free to refuse any question.

Nevertheless, our study has numerous strengths which support the validity of our findings. The data available on substance use within homeless populations is limited, and whilst our findings cannot be extrapolated to pre- or post-pandemic periods, factors associated with substance use may remain relevant. Moreover, the inclusion of multiple different substances within the same sample provides a particularly valuable, often-unreported comparison between substances with consistent demographics. Our study was conducted in two large metropolitan regions of France, thereby limiting the role of specific contextual factors on the selection of the homeless population living in accommodation centres. Finally, we interviewed participants who could not speak French or English, through professional interpreters, making it possible to collect data among recent migrants who constitute the largest share of the current homeless population in France.

### Conclusion

Our study shows elevated rates of substance use among homeless persons during the COVID-19 pandemic. Tobacco was the most commonly used substance, with rates roughly double that of the general population. The use of different substances also positively associated with each other, with the majority of users using more than one substance. Factors associated with tobacco and alcohol use varied, except being male and exposed to theft or assault, which increased the risk of using both substances. Higher rates of alcohol use were seen in the later stage of the pandemic, which may associate with participants’ greater housing insecurity during this wave. Together these findings provide valuable information on those most vulnerable to substance abuse within homeless populations. Considering the increased risk for both alcohol and tobacco-related injury amongst vulnerable persons, adapting interventions to better prevent substance use amongst those experiencing homelessness in the context of the COVID-19 pandemic is a public health necessity.
