# Supplementary material for: Substance Use Among Residents of Homeless Shelters During the COVID-19 Pandemic: Findings From France
Source: Int J Public Health. 2022 Aug 25;67:1604684. doi: 10.3389/ijph.2022.1604684 (PMC9452639; doi:10.3389/ijph.2022.1604684)
Supplement: Supplementary file 1 [file Table1.pdf]

SUPPLEMENTARY TABLE 1 | Description of the regional categories used for the ECHO study population (France, 2020-2021).

Category	Countries relevant to ECHO
France	France
Europe excluding France	Albania, Armenia, Belgium, Chechnya, Croatia, England, Georgia, Italy, Israel, Kosovo, Macedonia, Poland, Portugal, Romania, Russia, Serbia, Spain, Tajikistan, Ukraine, Yugoslavia
The Middle East	Afghanistan, Iran, Iraq, Pakistan, Palestine, Saudi Arabia, Somalia, Sudan
North Africa	Algeria, Egypt, Libya, Morocco, Tunisia
Sub-Saharan Africa	Central African Republic, Angola, Benin, Burkina Faso, Burundi, Cameroon, Cape Verde, Chad, Comoros, Democratic Republic of Congo, Ivory Coast, Eritrea, Gabon, Gambia, Guadeloupe, Guinea, Mauritius, Kenya, Liberia, Madagascar, Mali, Mauritania, Mayotte, Niger, Nigeria, Rwanda, Sierra Leone, Senegal, Togo, Zaire
Other	Barbados, Brazil, Bangladesh, Colombia, India, Kurdistan, Sri-Lanka, United States, Haiti, Mongolia, Peru, Polynesia, Tibet
